# Brain astrocytoma misdiagnosed as anti-NMDAR encephalitis: a case report

**DOI:** 10.1186/s12883-019-1436-x

**Published:** 2019-08-28

**Authors:** Jie Lu, Ji-hong Zhang, Ai-liang Miao, Jun-xiong Yin, Dong-lin Zhu, Xing-jian Lin, Dao-wen Chen, Jing-ping Shi

**Affiliations:** Department of Neurology, the Affiliated Brain Hospital of Nanjing Medical University, Nanjing, Jiangsu 210029 People’s Republic of China

**Keywords:** Anti-N-methyl-D-aspartate (anti-NMDA) receptor encephalitis, Anti-NMDAR antibody, Brain astrocytoma, Case report

## Abstract

**Background:**

Anti-N-methyl-D-aspartate receptor (anti-NMDAR) encephalitis, which is the most common type of autoimmune encephalitis, is caused by the production of autoantibodies against NMDA receptor. Anti-NMDAR encephalitis patients present with various non-specific symptoms, such as abnormal psychiatric or behaviour, speech dysfunction, cognitive dysfunction, seizures, movement disorders, decreased level of consciousness, and central hypoventilation or autonomic dysfunction.

**Case presentation:**

A 67-year-old man presented with new-onset focal seizures. The brain magnetic resonance imaging (MRI) plain scan and enhanced scan showed abnormal signal on the proximal midline frontoparietal junction region. Anti-NMDAR antibody was detected in cerebrospinal fluid (CSF) and serum using a commercial kit (Euroimmune, Germany) by indirect immunofluorescence testing (IIFT) according to the manufacturer’s instructions for twice. Both of the test results were positive in CSF and serum. The patient was diagnosed as anti-NMDAR encephalitis and then was treated repeatedly with large dose of intravenous corticosteroids and gamma globulin. Accordingly, the refractory nature of seizures in this case may be attributed to NMDAR autoantibodies. When the patient presented at the hospital for the third time, the brain MRI revealed an increase in the size of the frontal parietal lesion and one new lesion in the left basal ganglia. The patient underwent a surgical biopsy and astrocytoma was confirmed by histopathology.

**Conclusions:**

Although the sensitivity and specificity of anti-NMDAR-IgG antibodies in CSF to diagnose anti-NMDAR encephalitis are close to 100%, it is not absolute. Anti-NMDAR antibodies were positive, which might make the diagnosis more complex. The diagnosis of atypical presentation of anti-NMDAR encephalitis requires reasonable exclusion of other disorders.

## Background

Limbic encephalitis (anti-NMDAR encephalitis) was first identified in 2005 in four young women suffered from ovarian teratoma [1]. In 2007, anti-NMDAR encephalitis, firstly described by Dalmau and colleagues [2], is an acute disorder which presents a multistage illness progressing from memory disturbances to psychiatric symptoms, seizures, catatonia and dyskinesia. Anti-NMDAR encephalitis is a treatable [3] but often misdiagnosed autoimmune encephalitis. In the CSF or serum of patients, one can find antibodies produced by the body’s own immune system attacking NMDA receptors. Anti-NMDAR-IgG detection has been used as an important basis for the diagnosis of anti-NMDAR encephalitis, especially in CSF [4–7]. However, not all positive NMDAR-IgG antibodies in CSF and serum brought about the correct diagnosis of anti-NMDAR encephalitis.

We recently treated an elderly male patient presented with focal seizures, abnormal MRI signals limited to frontoparietal junction at the early stage of the disease. Anti-NMDAR antibody was detected in both the CSF and serum for twice. Both of the test results were positive in CSF and serum. The patient was diagnosed as anti-NMDAR encephalitis. Four months later, the patient underwent a surgical biopsy and histopathology revealed astrocytoma.

## Case presentation

The patient was a 67-year-old man with no significant medical history. He presented to the Nanjing Brain Hospital for the first time on July 4, 2016 with new onset frequent attacks of left limb convulsions without loss of consciousness nor incontinence for 6 days. The brain MRI from another hospital on June 30, 2016 showed abnormal signals in the left cingulate gyrus. During the hospitalization, the patient presented with frequent attacks (ten or more ictal attacks a day) of the left limb convulsions. Duration of attacks ranged from dozens of seconds to several minutes. There was no abnormality during the interval of the seizures. In the interictal period, the patient had no fever or headache, no mental or behavioral abnormalities, no dysphagia, no weakness of limbs, or other complications of nervous system.

**Fig. 1:**
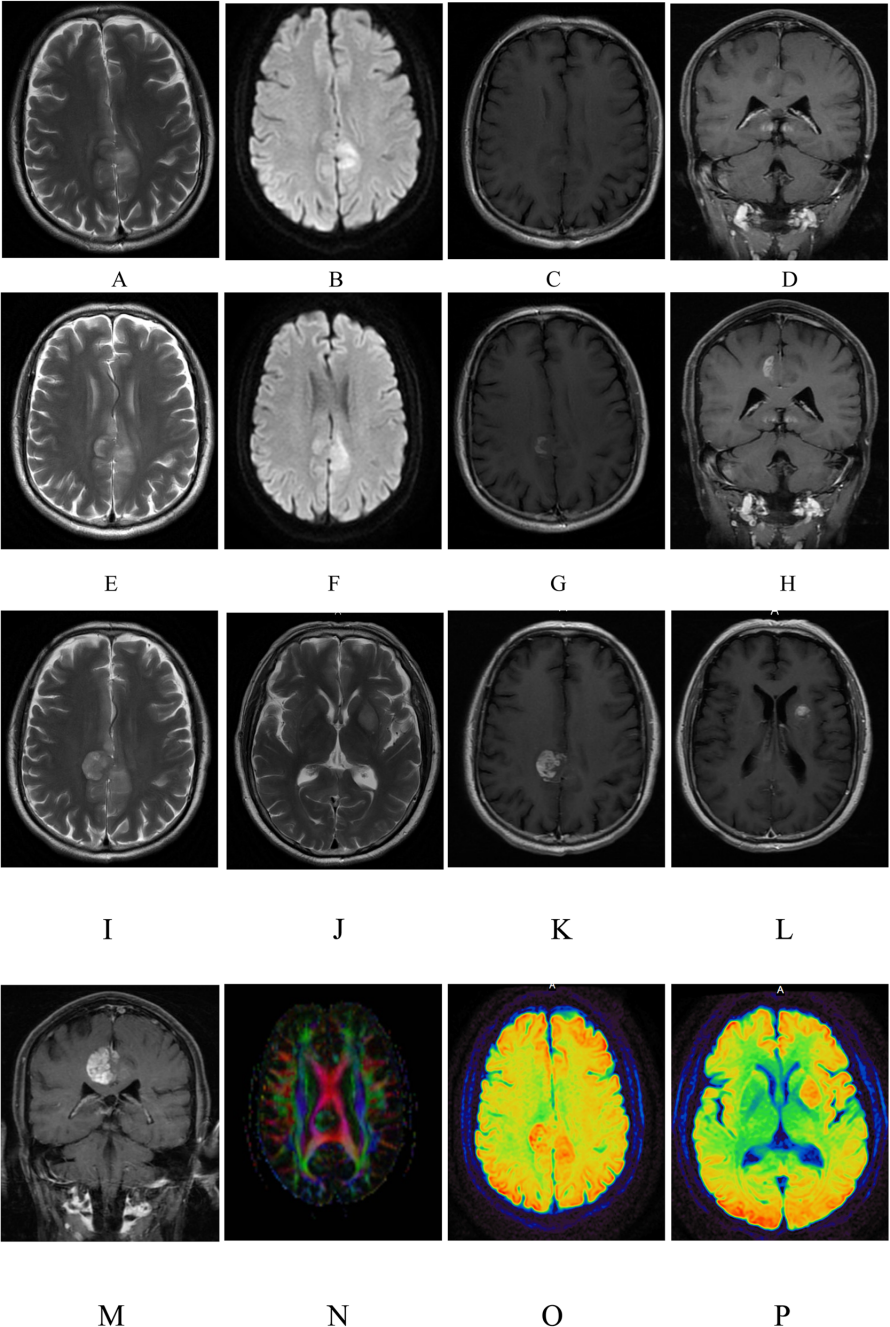
Brain MRI imaging of the patient. Brain MRI plain scan and enhanced scan (July 7, 2016) of the patient showed long T2 abnormal signal localized lesions (**a**) on the bilateral fronto parietal, proximal midline, Diffusion weighted imaging (DWI) high signal intensity (**b**), slight enhancement of lesions (**c**, **d**). Brain MRI plain scan and enhanced scan (September 18, 2016) of the patient showed bilateral fronto parietal, proximal midline, localized lesions long T2 signal(**e**), discrete wavelet transform (DWT) high signal high signal intensity (**f**), enhancement of lesions (**g**, **h**). Brain MRI plain scan and enhanced scan (November 15,2016) of the patient showed bilateral frontal and parietal lobes near the midline and left basal ganglia lesions long T2 signal(**i**, **j**), enhanced two lesions showed obvious enhancement (**k**, **l**, **m**), brain DTI showed partial fibrous bundle ablation of the bilateral frontal parietal lobe and left basal ganglia lesion. Fractional anisotropy (FA) decreased around the fiber bundle of mild compression and displacement(N). Arterial Spin Labeling (ASL): bilateral fronto parietal, left basal ganglia lesions were high perfusion (**o**, **p**)

Routine laboratory studies including blood and urine routine tests, coagulation tests, liver and renal function, blood sugar, glycosylated hemoglobin, antinuclear antibody, erythrocyte sedimentation rate, anti-cardiolipin antibodies, phospholipase A2, thyroid function, HIV and syphilis, were all unremarkable. Anti-glutamic acid decarboxylase (GAD) antibody was negative. Serum carbohydrate antigen 72–4 was 17.56 IU / ml (normal < 6.00 IU / ml), more than normal. Lumbar puncture revealed the CSF pressure of 100 mmH_2_O. Examination of the CSF showed white blood cells of 4/μl, protein levels of 0.45 g/L (normal 0.2 ~ 0.4 g / L). The concentrations of glucose and chlorine in the CSF were normal. Anti-NMDAR antibodies were detected in CSF and serum using a commercial kit (Euroimmune, Germany) by indirect immunofluorescence testing (IIFT) according to the manufacturer’s instructions for twice. Anti-NMDAR titers were 1:10(++) in CSF and 1:32(++) in serum. Anti-AMPA1, AMPA2, LG1, ASPR2 and GABA_B_ receptor antibodies in CSF and serum were negative. Tests for paraneoplastic antibodies (Hu, Yo, Ri, Ma2, CV2, Amphiphysin, ANNA-3, Tr, PCA-2, GAD) in CSF were all negative. Chest CT did not reveal any lesions concerning for malignancy. Video-EEG showed slight abnormality (all visible more low amplitude fast wave guide, especially the front head). Brain MRI scan and enhanced scan showed long T1 and long T2 abnormal signal on the bilateral frontal parietal, proximal midline, diffusion weighted imaging (DWI): high signal intensity, patchy eccentric mild enhancement (Fig. 1: a-d). MRS showed N-acetyl aspartate (NAA) peak decreased and no increase in choline compounds (Cho) peak. Therefore, lesions were considered the possibility of non-neoplastic lesions.

With a presumed diagnosis of anti-NMDAR encephalitis, the patient was treated with intravenous dexamethasone 10 mg/d × 11d → methylprednisolone 200 mg/d × 3d → methylprednisolone 80 mg/d × 3d → gamma globulin (20 g/d × 5d), oral prednisolone acetate tablets (40 mg qd) and antiepileptic (oxcarbazepine 300 mg bid). The epileptic seizures were slightly alleviated after treatment. The main manifestation was the twitching with spasms of the left upper and lower extremities, each lasting several seconds to more than ten seconds, attacking several times a day, without alteration of consciousness. Then the patient was discharged home.

Two months later, the patient presented to the hospital for a second time. He had suffered from frequent attacks of the left limb. Physical examination showed no abnormalities in the nervous system. Results from routine biochemical and cytological examination of the cerebrospinal fluid were normal. Anti-NMDAR titers were 1:32(+++) in CSF and 1:32(++) in serum. The brain MRI showed bilateral frontal parietal lesions enlarged slightly and the enhancement became more obvious than before (Fig.1 e-h). The patient was treated for a second time with intravenous injection of methylprednisolone (80 mg/d × 15d) → oral prednisolone acetate tablets (40 mg qd), immunoglobulins (25 g/d × 5d), oral azathioprine (50 mg qd), oral antiepileptic (oxcarbazepine 600 mg bid, Debakin 250 mg qm, 500 mg qn) treatment. The epileptic seizures were slightly alleviated after the treatment, 2~3 times a day, the twitching with spasms of the left limb typically lasts a few seconds, without loss of consciousness. Then the patient was discharged home.

**Fig. 2:**
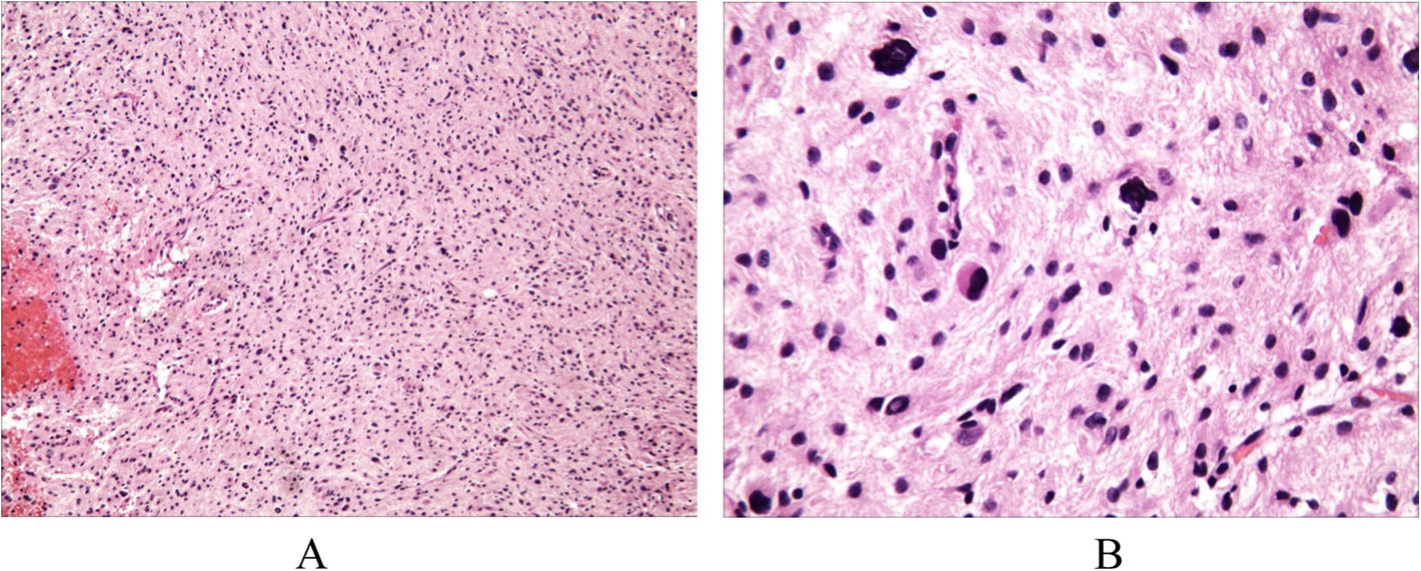
Histopathologic examination of biopsy lesions with hematoxylin–eosin staining. The tumor cells were diffusely distributed, densely packed, and rich in glial fibers. The tumor cells were of different sizes and abundant cytoplasm. The nuclei were round, oval and slender, with obvious atypia. The tumor tissues were rich in blood vessels without obvious tube wall proliferation, no necrosis area. **a**: The distribution of tumor cells was diffuse and the size of the tumor cells was different (HE × 100); **b**: The nuclei of the tumor cells were round, oval and slender, with obvious atypia (HE × 400)

Four months later, the patient presented at the hospital for a third time. He complained of weakness after frequent partial seizures in his right limb. Muscle Strength Grading Scale of the right lower extremity was 4/5 and physical examination of the remaining nervous system showed no significant abnormalities. The diagnosis was simple partial status epilepticus and Todd’s Paralysis. Epilepsy was controlled on diazepam injection therapy and the muscle strength of the right lower extremity returned to normal. The brain MRI reexamination indicated that the frontal parietal lesions enlarged and one new lesion appeared in the left basal ganglia with some mild mass effect, significantly enhanced (Fig.1 i-m). Diffusion tensor imaging (DTI) showed partial ablation of white matter fibro tracts in the bilateral frontal parietal lobe and the left basal ganglia lesion, reduction of FA and the surrounding fibro tracts slightly compressed (Fig.1 n). Arterial spin labeling (ASL) MR imaging showed that bilateral frontoparietal and left basal ganglia lesions had abnormally high perfusion (Fig.1 o-p). Surgical biopsy of the right parietal lesion was performed under general anesthesia on November 21, 2016. The lesions were pale red, slightly tough, abundant blood supply and without clear border. The size of the removed lesion was about 1.5 × 1.5 × 1.0 cm. The astrocytoma (WHO II-III) (Fig. 2) was confirmed on histopathologic findings. Patients transferred to the oncology department for further radiotherapy and chemotherapy. One month later, the patient died.

## Discussion and conclusions

Anti-NMDAR encephalitis is a type of autoimmune encephalitis, which is induced by anti-NMDAR subunit NR1 antibodies [7–9]. Anti-NMDAR encephalitis was initially reported as a paraneoplastic syndrome associated with ovarian teratoma [10]. However, it is now acknowledged that the spectrum of this encephalitis is much broader, as there have been many cases in women without ovarian teratoma, men, and children [3]. Typical patients with anti-NMDAR encephalitis show nonspecific prodromal symptoms, such as a fever and headache, followed by symptoms can resembling schizophrenia, with subsequent development of generalized seizure, altered mental status, hypoventilation, autonomic instability, and characteristic movement disorders, such as orofacial-limb dyskinesia and catatonia [2, 3, 8]. Brain MRI is unremarkable in 50% of patients [2, 11], FLAIR signal hyperintensity or T_2_ might be seen in the hippocampi, cerebral cortex or cerebellar, insular and frontobasal regions, basal ganglia, brainstem and the spinal cord [8, 12]. Earlier reports of patients who did not recover or died, or who had refractory seizures, showed significant brain atrophy [9]. Lesions may occur that appear demyelinating, are transient, and do not usually enhance. The EEG may be useful for diagnosis of anti-NMDAR encephalitis [5, 13]. EEG usually shows non-specific, slow, and disordered background activity. However, the EDB pattern in 2012 was recognized as a unique EEG pattern in some anti-NMDAR encephalitis patients [5, 13]. In 80% of patients, the CSF is initially abnormal and becomes abnormal later. Typical clinical and other characteristics include normal or mildly increased protein concentration, moderate lymphocytic pleocytosis and CSF-specific oligoclonal bands in 60% of patients [8, 12]. The NMDAR antibodies were intrathecal synthesized in most patients. Antibody testing should be performed in serum and CSF. Although clinical manifestations and auxiliary examination results of imaging, CSF and EEG support the diagnosis of anti-NMDAR encephalitis, the diagnosis depends on the detection of anti-NMDAR-IgG antibodies in serum and CSF, especially in CSF, and other possible conditions with similar symptoms need to be excluded.

The introspection on the diagnosis and treatment process of this case, early symptoms of this patient were simple partial seizures and the MRI showed abnormal signals in bilateral frontoparietal junction regions with no obvious enhancement. Anti-NMDAR was positive in CSF and serum. Therefore, the initial diagnosis of “anti-NMDAR encephalitis” was understandable. Epilepsy was poorly controlled on immunotherapy at the first hospitalization. The brain MRI lesions volume at the second hospitalization had increased and the diagnosis of “anti-NMDAR encephalitis” was suspected. However, once again review of serum and CSF anti-NMDAR-IgG were still positive. The diagnosis of anti-NMDAR encephalitis was not unreasonable. Until four months later, MRI showed lesions of the “tumor appearance”. Therefore, the detection of antibodies is a key step in the definite diagnosis of multiple autoimmune encephalitis. Clinicians must be aware of the potential pitfalls in interpreting the results. Treatment decisions should rely more on clinical assessment than on antibody test.

Epileptic seizures are reported to be one of the common symptoms of anti-NMDAR encephalitis, however epilepsy as the presenting symptom in male patients is relatively common [8, 14]. During the first month of the anti-NMDAR encephalitis, 87% (498 /571) patients presented four or more of the 8 categories of symptoms [12]; only 1% (6/571) remained mono-symptomatic. Large sample study of anti-NMDAR encephalitis cases (577 cases) data showed 81% of these patients were young female [14] with only 5% of them being 45 years or older. The eight symptom categories of anti-NMDAR encephalitis [14, 15] are: memory dysfunction, behavior/cognition, seizures, speech disorder/mutism, decrease in level of consciousness, autonomic dysfunction, movement disorder and central hypoventilation. The brain MRI, EEG and CSF examinations were abnormal [14] in 33%(180/540), 90% (432/482) and 79% (418/532) patients, respectively. In contrast, this case, the elderly patients with no history of cancer, the clinical manifestation was only epileptic seizures, CSF examination without inflammatory changes, large dose of corticosteroids and gamma globulin was ineffective. These clinical features were untypical or unsupportive of the evidence for the diagnosis of anti-NMDAR encephalitis.

Clinical studies [13–16] have shown that only some of the cases of anti-NMDAR encephalitis are associated with tumors, and the most common is ovarian teratoma. Anti-NMDAR encephalitis has been recognized in all age groups, but is more common in young adults and children with or without teratoma. Studies have reported [17] that 38% (220) patients had an underlying neoplasm; 213 of these were women. Tumors predominated in patients aged 12–45. The 94% tumors (207) were ovarian teratomas [17], 2% (4) extraovarian teratomas, and 4% (9) other tumors (lung, breast, testicular; ovarian carcinoma and so on). To our knowledge, no data to date are available about anti-NMDAR IgG positive is associated with brain astrocytoma. It should be noted that serum anti-NMDAR-IgG positive also exists in the general population and other neuropsychiatric patients (schizophrenia, infectious diseases, stroke, Parkinson’s disease, amyotrophic lateral sclerosis, and personality disorders), but the positive rate is only about 1% [6]. Although this case was ultimately diagnosed as astrocytoma, it is unclear that the anti-NMDAR-IgG positive in serum and CSF is directly related to astrocytoma. At the same time, we can not exclude the possibility that NMDAR antibodies were relevant to this patient’s clinical presentation.

Glioma is a common primary tumor of the central nervous system and lacks specific clinical manifestations. In other words, its imaging changes are also varied, which will lead to a rate of misdiagnosis. The diagnosis is more often dependent on the results of a diagnostic brain biopsy. Epileptic seizures are common clinical symptoms of glioma, often the early symptom. Glioma may be considered in this case when the patient was first admitted to the hospital, but the early image changes do not support. Although the sensitivity and specificity of detection of anti-NMDAR-IgG antibodies in CSF to diagnose anti-NMDAR encephalitis are close to 100% [7, 16] and the clinical manifestations of anti-NMDAR encephalitis and routine CSF, imaging and EEG examination have certain specificity, it is not absolute. The diagnosis of an atypical presentation of anti-NMDAR encephalitis should require reasonable exclusion of other disorders. If necessary, we should perform a stereotactic brain biopsy so as not to delay of diagnosis and implement unnecessary immunotherapy.

## Data Availability

Data sharing is not applicable to this article as no datasets were generated or analysed during the current study.

## Abbreviations

Anti-NMDAR: Anti-N-methyl-D-aspartate receptor
ASL: Arterial spin labeling
Cho: Choline compounds
CSF: Cerebrospinal fluid
DWI: Diffusion weighted imaging
EDB: extreme delta brush
EEG: Electroencephalogram
GAD: Glutamic acid decarboxylase
IIFT: Indirect immunofluorescence testing
MRI: Magnetic resonance imaging
NAA: N-acetyl aspartate
